# Diabetic hyperglycemia attenuates sympathetic dysfunction and oxidative stress after myocardial infarction in rats

**DOI:** 10.1186/s12933-014-0131-x

**Published:** 2014-10-10

**Authors:** Christiane Malfitano, Catarina Andrade Barboza, Cristiano Mostarda, Renata Kelly da Palma, Camila Paixão dos Santos, Bruno Rodrigues, Sarah Cristina Ferreira Freitas, Adriane Belló-Klein, Susana Llesuy, Maria-Cláudia Irigoyen, Kátia De Angelis

**Affiliations:** Translation Physiology Laboratory, Universidade Nove de Julho (UNINOVE), Vergueiro 235/249 – 2° subsolo – Mestrado, Zip Code 01504 001 São Paulo, SP Brazil; Human Movement Laboratory, Universidade São Judas Tadeu UST, São Paulo, SP Brazil; Universidade Federal do Maranhão (UFMA), São Luís, MA Brazil; Hypertension Unit, Heart Institute (InCor), Medical School of University of São Paulo, São Paulo, SP Brazil; Laboratório de Fisiologia Cardiovascular e Espécies Ativas de Oxigênio, Universidade Federal do Rio Grande do Sul, Porto Alegre, Brazil; Cátedra de Química General y Inorgánica, Facultad de Farmácia y Bioquimica, Universidad de Buenos Aires, Buenos Aires, Argentina

**Keywords:** Autonomic modulation, Oxidative stress, Diabetic hyperglycemia, Myocardial infarction

## Abstract

**Background:**

Previous research has demonstrated that hyperglycemia may protect the heart against ischemic injury. The aim of the present study was to investigate the association between hyperglycemia and myocardial infarction on cardiovascular autonomic modulation and cardiac oxidative stress profile in rats. Male Wistar rats were divided into: control (C), diabetic (D), myocardial infarcted (MI) and diabetic infarcted rats (DMI).

**Methods:**

Diabetes was induced by streptozotocin (STZ, 50 mg/Kg) at the beginning of the protocol and MI was induced by left coronary occlusion 15 days after STZ. Thirty days after streptozocin-induced diabetes, cardiovascular autonomic modulation was evaluated by spectral analysis, and oxidative stress profile was determined by antioxidant enzyme activities and superoxide anion, together with protein carbonylation and redox balance of glutathione (GSH/GSSG).

**Results:**

The diabetic and infarcted groups showed decreased heart rate variability and vagal modulation (p < 0.05); however, sympathetic modulation decreased only in diabetic groups (p < 0.05). Sympatho/vagal balance and vascular sympathetic modulation were increased only in the MI group (p < 0.05). Diabetes promoted an increase in catalase concentration (p < 0.05). Glutathione peroxidase activity was increased only in DMI when compared to the other groups (p < 0.05). Superoxide anion and protein carbonylation were increased only in MI group (p < 0.05). Cardiac redox balance, as evaluated by GSH/GSSG, was lower in the MI group (p < 0.05).

**Conclusions:**

These data suggest that hyperglycemia promotes compensatory mechanisms that may offer protection against ischemia, as demonstrated by increased antioxidants, decreased pro-oxidants and protein damage, possibly related to the improvements in both redox balance and sympathetic modulation to the heart.

## Introduction

It is well established that cardiovascular autonomic neuropathy is a major complication of diabetes. It is a common manifestation in diabetics and contributes to the overall clinical pathology [1,2]. In addition, diabetic cardiomyopathy is a serious diabetes complication, which is functionally characterized by ventricular dilation, myocyte hypertrophy, and prominent interstitial fibrosis, along with decreased systolic function. The predisposing factors in diabetes are associated with a two – to four –fold increased risk of coronary heart disease [3].

Despite large body of clinical evidence indicating that diabetic hearts are more sensitive to ischemic injury, available data from experimental diabetic animal models are conflicting. Some studies have shown that the diabetic heart is less sensitive to ischemic injury [4-9] while others have suggested that the diabetic heart would be more sensitive [10-12]. Several factors have been proposed to account for such discrepancy. Our group has previously demonstrated that left ventricular dysfunction in chronic diabetic animals was attenuated after 90 days of myocardial infarction. This finding was associated with better profile of calcium handling proteins in left ventricle of diabetic infarcted rats [13]. Additionally, the activation of hypoxia-inducible factor (HIF-1) was correlated with hypoxia-induced angiogenesis before and 15 days after myocardial infarction induced a reduction of fibrosis in diabetic animals (30 days of diabetes) [7].

Furthermore, after myocardial infarction the blood flow in the heart is reduced, decreasing the substrates which are essential to myocardium workload. This pathological condition favors an imbalance between reactive oxygen species (ROS) production and the protective antioxidant defense system, thereby increasing ROS mediated oxidative stress [14]. Moreover, it is well established that increased oxidative stress plays a critical role in diabetes complications, as demonstrated by increased levels of oxidized DNA, proteins and lipids in diabetic subjects [15]. However, the role of ROS in the cardiac response of diabetic hearts to myocardial infarction remains unclear.

A fuller understanding of the mechanisms underlying ROS generation and reactivity may result in new intervention strategies, which might contribute to reduce the cellular damage associated with oxidative stress in diabetic hearts after ischemic injury. Therefore, the aim of the present study was to investigate the association between hyperglycemia and myocardial infarction in cardiovascular autonomic modulation and cardiac oxidative stress profile in rats undergoing 15 days of ischemia.

## Materials and methods

### Experimental design

All experimental procedures were conducted according to the Guidelines for the Use and Care of Animals Research issued by the National Institute of Health, and complied with the ARRIVE guidelines for reporting animal research [16]. The study protocol was approved by the Ethics Research Committee of Nove de Julho University (process N° 00013/2012). Experiments were performed using adult male Wistar rats (2–3 months old, 250–270 g) from the animal facility at the Nove de Julho University (São Paulo, Brazil). The rats were fed standard laboratory chow and water ad libitum. They were housed in collective polycarbonate cages in a temperature-controlled room (22°C) with a 12-hour darklight cycle. The rats were randomly assigned to 4 groups (n = 8, per group): control (C), diabetic (D), myocardial infarcted (MI), and diabetic myocardial infarcted (DMI) rats.

Diabetes (D and DMI groups) was induced on the first day of the experiments. On the 15^th^ day of protocol (15 days after diabetes induction), myocardial infarction was induced in both DMI and the MI groups. Body weight was evaluated weekly. All groups were assessed for 30 days and hemodynamic, cardiovascular autonomic modulation and oxidative stress evaluations were performed 30 days after buffer or STZ injection, and 15 days after MI, as previously described [7].

### Induction of diabetes

On the first day of the experiment a single injection of streptozotocin (STZ, 50 mg/kg, iv; Sigma Chemical Co., St. Louis, MO, USA, dissolved in citrate buffer, pH 4.5) was given to male adult rats (2–3 months old, 250-270 g, to induce diabetes (D and DMI groups) while C and MI rats received a single injection of citrate buffer. Blood glucose was measured to confirm the diabetic-induced hyperglycaemia at the beginning of the 30-day protocol after STZ injection (Accu-Check Instant test, Boehringer Mannheim).

### Induction of myocardial infarction

Fifteen days after diabetes induction or administration of citrate buffer, rats of MI and DMI groups were anaesthetized (80 mg/kg ketamine and 12 mg/kg xylazine, i.p.) and underwent induction of MI by surgical occlusion of the left coronary artery, as described elsewhere [7,17]. Briefly, left thoracotomy was performed, the third intercostal space dissected, and the heart exposed. The left coronary artery was occluded with a single nylon (6.0) suture at approximately 1 mm distal to the left atrial appendage. The chest was closed with silk suture. The animals were maintained under ventilation until recovery. Animals in the (C) and (D) groups underwent sham surgery without induction of myocardial infarction.

### Myocardial infarction size

Myocardial infarction size was estimated on the 16th day of protocol (one day post myocardial surgery) and at the end of protocol (30^th^ of protocol) on the basis of subjective identification of akinesia or dyskinesias. MI size % of left ventricle (LV) perimeter was measured as the ratio of these regions to the total perimeter of the endocardial border of the LV. To confirm the echocardiography measurement, the MI size was also evaluated by dissecting the fibrous scar from the remaining LV muscle [7].

### Heart rate and arterial pressure variabilities

One day after the final echocardiographic evaluation (31 days of protocol) two catheters filled with 0.06 ml of saline were implanted; one into the right carotid artery to capture blood pressure (BP) signals, and a second one into the jugular vein for saline administration of each anesthetized rat (80 mg/Kg ketamine and 12 mg/Kg xylazine, i.p). Twenty-four hours later (32 days of protocol), the arterial catheter was connected to a strain gauge transducer (Blood Pressure XDCR; Kent Scientific, Torrington, CT, USA), and BP signals were recorded over a 30 minutes period in conscious animals using a microcomputer equipped with an analog-to-digital converter board (WinDaq, 2 kHz, DATAQ, Springfield, OH, USA). The recorded data were analyzed on a beat-to-beat basis to quantify changes in the systolic (SBP), diastolic (DBP) and mean BP (MAP) and heart rate (HR).

The total power of heart rate variability (HRV) and systolic blood pressure variability (BPV) were evaluated using BP recordings obtained in conscious rats at rest (continuous 30 minutes, 2000 Hz). Overall variability of HR and SBP were assessed in the time domain by means of variance. HR and SBP fluctuations were assessed in the frequency domain by using autoregressive spectral analysis, as described elsewhere [18]. Briefly, HR and SBP series were divided into segments of 350 beats and overlapped by 50%. A spectrum was obtained for each of the segments via the Levinson-Durbin recursion, with the model order chosen according to Akaike's criterion, ranging between 10 and 14. The oscillatory components were quantified in low (LF: 0.2 to 0.75 Hz) and high (HF: 0.75 to 3.0 Hz) frequency ranges [19]. The power spectrum density was calculated for each recognizable component in the LF and HF bands by integrating the spectrum of the components. The power is expressed as LF and HF power, as described elsewhere [20].

### Oxidative stress profile

After cardiovascular evaluations, on the 32^nd^ day of protocol the animals were euthanized by decapitation; the left ventricle (LV) was rapidly removed, washed in saline phosphate buffer (PBS), weighed and frozen at −70°C for oxidative stress analyses.

Approximately ~100 mg of the LV apices were used for evaluation of the GSH/GSSG. The remaining LV (ratio of 1 gram of tissue/5 ml of 150 mmol/L of KCl and 20 nmol/L of phosphate buffer, pH 7.4, with 20 mM phenylmethylsulfonyl fluride proteases inhibitor solution) was cut into small pieces, placed in ice-cold buffer and homogenized using an Ultra Turrax blender. The LV homogenate was then centrifuged at 600 g for 10 minutes at 2°C to remove nuclei and cell debris, as described elsewhere [21]. The protein content of LV homogenate was measured by the Lowry method [22], using bovine serum albumin as the standard.

### Antioxidant enzymes

Superoxide dismutase (SOD) activity was measured spectrophotometrically in LV homogenates by rate inhibition of pyrogallol autooxidation at 420 nm [23]. Enzyme activity was reported as U/mg protein. Catalase (CAT) activity was determined in LV homogenates by measuring the decreased absorbance (240 nm) of hydrogen peroxide (H_2_O_2_). The results are expressed as nmol of reduced H_2_O_2_/min/mg protein [24]. Glutathione peroxidase (GPx) activity was assessed in LV homogenates by adding to the assay a mixture of 1 U/mL glutathione reductase and 2 mmol/L glutathione in 1 mL phosphate buffer. Mixtures were preincubated at 37°C for 30 minutes. Subsequently, NADPH and tert-butylhydroperoxide were added, and the change in absorbance at 340 nm was recorded to calculate GPx activity, as previously described [25,26].

### Superoxide anion

The superoxide anion was determined in LV homogenates by calculating the rate of oxidation of adrenaline at 480 nm, as described by McCord and Fridovich [27]. Since other reactive oxygen species than anion superoxide can also oxidize adrenaline, we added SOD to the assay and observed a critical inhibition of the reaction, suggesting that this anion might play the most influential role in this assay.

### Protein carbonylation

This method uses the a reaction of protein carbonyl groups with 2,4- *dinitro phenylhydrazine* (DNPH) to form a 2,4-dinitrophenylhydrazone. The product of the reaction was measured at 360 nm, as previously described [28]. The concentration of the carbonyl in LV homogenates was standardized on the protein unit (nmol carbonyl group/mg protein) in homogenates of LV. The amount of protein was calculated from the bovine serum albumin dissolved in guanidine hydrochloride and read at 280 nm. Results were expressed as nmDNPH/mg protein.

### Thiobarbituric acid reaction (TBARS)

For the TBARS assay, trichloroacetic acid (10%, w/v) was added to the LV homogenates to precipitate proteins and to acidify the samples [29]. This mixture was then centrifuged (10006 g, 3 minutes), the protein-free sample was extracted, and thiobarbituric acid (0.67%, w/v) was added to the reaction medium. The tubes were placed in a water bath (100°C) for 15 minutes. The absorbances were measured at 535 nm using a spectrophotometer. Commercially available malonyldialdehyde (MDA) was used as a standard, and the results are expressed as nmoles/mg protein.

### Determination of oxidized and reduced glutathione concentration

In order to determine the concentrations of reduced form (GSH) and oxidized glutathione form (GSSG), the LV tissue (~100 mg) was deproteinized with 2 mol/L perchloric acid and centrifuged (1000 g, 10 minutes) while the supernatant was neutralized with 2 mol/L potassium hydroxide. The reaction medium contained 100 mmol/L phosphate buffer (pH 7.2), 2 mmol/L nicotinamide adenine dinucleotide phosphate (NADPH), 0.2 U/mL glutathione reductase, and 70 mmol/L 5, 50 dithiobis (2-nitrobenzoic acid). To determine reduced glutathione, the supernatant was neutralized with 2 mol/l potassium hydroxide to react with 70 mmol/L 5, 50 dithiobis (2-nitro benzoic acid), and absorbance values were measured at 420 nm [30].

### Statistical analysis

Data are reported as means ± SEM. After confirming that all continuous variables were normally distributed using the Kolmogorov-Smirnov test. One-way or two-way ANOVA followed by the Student-Newman-Keuls *post-hoc* test was used to compare groups. Pearson correlation was used to study the association between variables. Differences were considered significant at p ≤ 0.05 for all tests. Calculations were performed with Statistical Package for Social Sciences (SPSS) software, version 12.0.

## Results

### Animals

At the beginning of the protocol, body weight was not statistically different between study groups (C: 280 ± 17; D: 298 ± 10; MI: 313 ± 4; DMI: 271 ± 4.5 g). Diabetes induced decreases in body weight at the end of the experimental protocol (D: 297 ± 15 g and DMI:309 ± 10 g) when compared to the non-diabetic groups (C: 400 ± 10 and MI: 394 ± 13 g). Furthermore, the STZ-diabetic rats (D: 415 ± 22 and DMI: 385 ± 25 mg/dL) had higher plasma glucose levels when compared to the normoglycemic rats (C: 88 ± 4 and MI: 92 ± 3 mg/dL).

Interestingly, at the end of protocol the akinetic area in the diabetic group was smaller (DMI: 24 ± 3% of LV perimeter) when compared to the MI group (39 ± 2% of LV perimeter).

### Hemodynamic measurements

SBP remained the same for all groups (C: 123 ± 6; D: 112 ± 4; MI: 122 ± 6; DMI: 111 ± 3 mmHg). DBP was decreased after diabetes and myocardial infarction (D: 82 ± 2; MI: 82 ± 2; DMI: 89 ± 3 mmHg) when compared to controls (96 ± 2 mmHg). MBP was reduced in diabetic group (D: 92 ± 2 mmHg) when compared to control group (C: 105 ± 2 mmHg), and was not altered after myocardial infarction (MI: 95 ± 3 and DMI: 96 ± 3 mmHg).

HR was increased after myocardial infarction (MI: 358 ± 4 and DMI: 358 ± 13 bpm) when compared to D and C groups (316 ± 9 and 330 ± 10 bpm, respectively).

### Heart rate and blood pressure variability

Table 1 shows that the D, MI and DMI groups present decreases in heart rate variance and vagal modulation (the absolute power of the HF) when compared to C group. The diabetic groups (D and DMI) promoted a decrease in sympathetic modulation to the heart (the absolute power of LF) when compared to C and MI groups. However, the normalized power of HF component of HRV was decreased in the MI group when compared to C, D and DMI groups, while the normalized power of the LF component of HRV was increased in the MI group when compared to C, D and DMI groups. Therefore, the sympathovagal balance (LF/HF) was increased only in the MI group when compared to the other studied groups.

**Table 1:** **Cardiovascular autonomic modulation in studied groups**

**Measurements**	**C**	**D**	**MI**	**DMI**
***HRV***				
**Variance (ms** ^**2**^ **)**	122 ± 15	35 ± 4.0*	39.5 ± 6.0*	23 ± 2.5*
**LF (ms** ^**2**^ **)**	3.0 ± 0.28	1.3 ± 0.36 *^†^	4.0 ± 0.53	1.2 ± 0.10*^†^
**LF (NU)**	20 ± 1	14 ± 3^†^	36.3 ± 3.2*	12 ± 1.6^†^
**HF (ms** ^**2**^ **)**	12 ± 1.0	6.7 ± 0.7*	5.3 ± 0.9*	6. ± 0.6*
**HF (NU)**	80 ± 1	86 ± 3^†^	64 ± 3.2*	88 ± 1.6^†^
**LF/HF**	0.26 ± 0.02	0.11 ± 0.02*^†^	0.40 ± 0.04*	0.14 ± 0.02*^†^
***BPV***				
**Variance (mm Hg** ^**2**^ **)**	28 ± 4.0	9 ± 1.3*^†^	49 ± 7.5*	2 ± 0.3*^†^
**LF (mm Hg** ^**2**^ **)**	2.5 ± 0.80	1.0 ± 0.16*^†^	6 ± 0.53*	1.0 ± 0.09*^†^

Systolic blood pressure (BPV) and heart rate (HRV) variabilities computed from 0.20 to 3 Hz (total power), low-frequency (LF: 0.20-0.75 Hz) and high-frequency (HF: 0.75-3 Hz) bands of control (C), diabetic (D), myocardial infarcted (MI) and diabetic myocardial infarcted (DMI) rats. Data are reported as mean ± SEM. *p ≤ 0.05 compared with C; ^†^p ≤ 0.05 compared with MI.

In addition, vascular sympathetic modulation (LF band) was increased in MI group in relation to the other groups (Table 1). Systolic BP variance was decreased in diabetic groups (D and DMI) when compared to the C group; however, this parameter was increased in the MI group when compared to the other studied groups (Table 1).

### Oxidative stress profile

**Figure 1:**
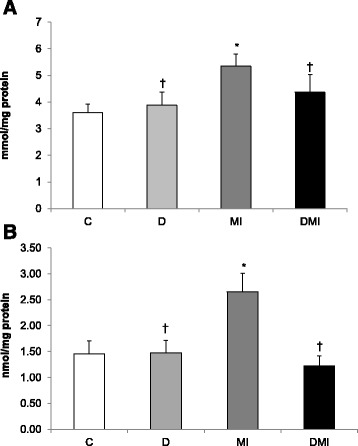
**Oxidative stress profile of left ventricle homogenates in control (C), diabetes (D), myocardial infarction (MI), and diabetes + myocardial infarction (DMI) rats (n = 8 for each group). A**: Lipid peroxidation indicated by thiobarbituric acid-reactive substances (TBARS). **B**: protein carbonylation. *p < 0.05 vs. C; ^†^p < 0.05 vs. MI.

**Figure 2:**
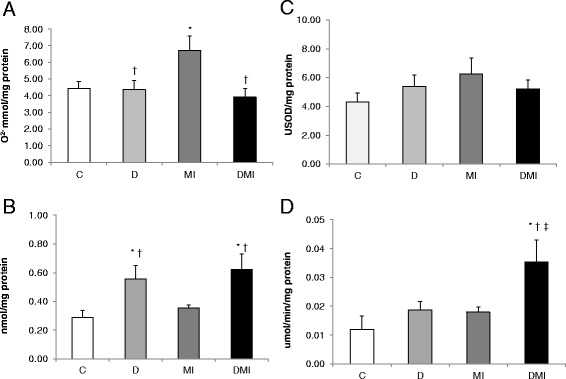
**Oxidative stress profile of left ventricle homogenates in control (C), diabetes (D), myocardial infarction (MI), and diabetes + myocardial infarction (DMI) rats (n = 8 for each group). A**: superoxide anion. **B**: catalase activity. **C**: superoxide dismutase activity. **D**: glutathione peroxidase activity. *p < 0.05 vs. C; ^†^p < 0.05 vs. MI; ^‡^p < 0.05 vs. D.

Myocardial infarction induced an increase in cardiac membrane lipid peroxidation, as assessed by TBARS (MI: 6 ± 0.4 vs. D: 4 ± 0.5; DMI: 4 ± 0.5 and C: 4 ± 0.3 mmol/mg protein) (Figure 1A), and increased protein carbonylation (MI: 2.65 ± 0.36 vs. D: 1.5 ± 0.24; DMI: 1.22 ± 0.2 and C: 1.45 ± 0.26 nmol/mg protein) when compared to the other studied groups (Figure 1B). Increases in these parameters of damage were not found in either D or DMI groups. In addition, the levels of superoxide anion increased in the MI group when compared to the others groups (MI: 7 ± 0.9 vs. D: 4 ± 0.5; DMI: 4 ± 0.5 and C: 4 ± 0.4 O^2−^ mmol/mg protein) (Figure 2A).

These changes were accompanied by significant alterations in cardiac antioxidant enzymes. Diabetes promoted an increase in CAT activity (DMI: 0.62 ± 0.1; D: 0.56 ± 0.09 vs. IM: 0.35 ± 0.02 and C: 0.29 ± 0.05 nmol/mg protein) (Figure 2B). However, GPx activity was increased only in the DMI group (0.04 ± 0.008 μmol/min/mg protein) when compared to the other groups (MI: 0.02 ± 0.0002; D: 0.02 ± 0.003; and C: 0.01 ± 0.005 μmol/min/mg protein) (Figure 2C). SOD activity was not statistically different between groups (C: 4.32 ± 0.62; D: 5.38 ± 0.82; MI: 6.27 ± 1.1 and DIM: 5.22 ± 0.63 USOD/mg protein) (Figure 2D).

**Figure 3:**
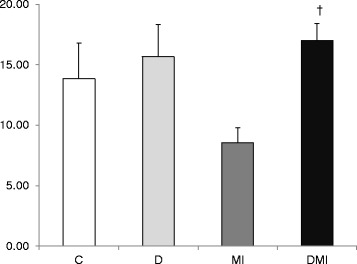
**Oxidized (GSH) and reduced glutathione (GSSG) ratio in left ventricle tissue in control (C), diabetes (D), myocardial infarction (MI), and diabetes + myocardial infarction (DMI) rats (n = 8 for each group).** ^†^p < 0.05 vs. MI.

The GSH/GSSG ratio was reduced only in MI group (8.5 ± 1.2) when compared to the other studied groups (C: 14 ± 3; D: 16 ± 3; DMI: 17 ± 1.4) (Figure 3).

### Correlation analysis

**Figure 4:**
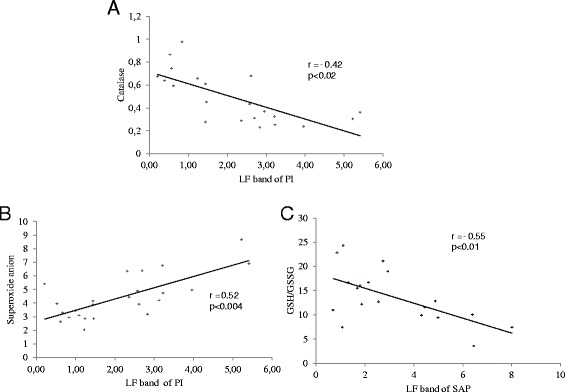
**Pearson correlation analysis involving animals of all groups (C; D; MI and DMI). A**: Cardiac sympathetic modulation (LF band of PI) and catalase. **B**: Cardiac sympathetic modulation (LF band of PI) and superoxide anion. **C**: Vascular sympathetic modulation (LF band of SAP) and GSH/GSSG ratio.

Correlations between cardiac sympathetic modulation (LF band) and CAT (r = − 0.68; p < 0.0006, Figure 4A), and superoxide anion (r = 0.7; p < 0.0001, Figure 4B.) were found using Pearson correlation analysis and involved all groups. This indicates that higher values of LF band were associated with both lower CAT concentration and higher levels of superoxide anion. Moreover, a significant inverse correlation between vascular sympathetic modulation (LF band) with cardiac GSH/GSSG ratio (r = − 0.60; p < 0.05, Figure 4C.) was also found, with higher vascular LF band being associated with lower glutathione redox balance.

## Discussion

Hyperglycemia leads to abnormalities in several organs and these damages in turn increase the morbidity and mortality in diabetic individuals. However, some studies have suggested that hyperglycemia may offer a protective effect against myocardial ischemia, but these findings remain controversial. To understand the positive role of hyperglycemia on gene regulators of cell survival, the present study investigated the effects of 15 days of myocardial infarction on cardiac autonomic modulation and on oxidative stress profile in STZ-diabetic rats (30 days). The key finding of the present study lies in the fact that the myocardial infarction induced increase in cardiac and vascular sympathetic modulation was attenuated in diabetic infarcted rats, and this change was associated with improvement in cardiac oxidative stress profile.

The present study corroborates a previous study undertaken by our group; in that study we found a similar metabolic damage induced by STZ diabetes [31-33]. The diabetic groups in this study presented a statistically significant increase in glycemia and reduction in body weight, therefore demonstrating a significant metabolic state which closely resembles humans with uncontrolled diabetes. Interestingly, some studies [34-36] have reported conflicting data about the role of hyperglycemia in the mechanisms that would account for cardioprotection in diabetic animals. Researchers have observed a pronounced reduction in the myocardial infarction area in diabetic animals, which may be a result of increased availability and use of glucose, the heart’s preferred energy substrate during a stress [9,37,38]. These studies showed that the hyperglycemic conditioning before ischemia leads to a cardioprotective condition; however, the underlying protective mechanisms should be further studied.

The leading cause of the higher resistance of diabetic rats to ischemic injury may be associated with the reduction in myocardial infarction size [39] promoted by the reduced fibrosis area and the number of dead myocytes [40]. These suggest an improvement in systolic function generated by decreased pro-inflammatory cytokines in diabetic animals [7].

Although diabetes worsens global left ventricular function, it protects the ischemic area, leading to increased expression of cell survival proteins and decreased infarct size [9]. In fact, in the present study we observed that after 15 days of myocardial infarction, diabetic animals presented a statistically significant reduction in the akinetic area, as previously described by our group (DMI animals compared to MI animals) [7]. This finding could be associated with an increase in survival pathways, such as anti-apoptotic factor (Bcl-2), protein kinase B (Akt), protein Kinase C-ε (PKC-ε), HIF-1 and vascular endothelial growth factor (VEGF). Accordingly, capillary density may effectively contribute to the reduction of ischemic injury and cardiac fibrosis in diabetic animals. These alterations might play an important role in improving heart function and increasing glucose transporter type 1 expression, which in turn may be critical for glucose uptake in ischemic conditions in diabetic infarcted rats [41].

### Hemodynamic and cardiovascular autonomic modulation

DBP was reduced in DMI group and this change in BP may be related to cardiovascular autonomic neuropathy, which has been regarded as a major determinant of mortality in diabetic patients, regardless of other known risk factors [42]. Indeed, in line with these findings, in the present study both the diabetic and infarcted groups showed a statistically significant decrease in heart rate variance and vagal modulation.

To further investigate the mechanisms underlying the improved tolerance to ischemia in diabetic rats (DMI group) we evaluated the cardiac autonomic modulation of the experimental groups. Interestingly, our data indicate that diabetes attenuated the sympathetic dysfunction caused by myocardial ischemia. The vascular and cardiac sympathetic modulations (LF bands), as well as the cardiac sympathovagal balance, were increased in MI when compared to DMI group (p < 0.05). Possibly, this cardiovascular autonomic dysfunction might lead to cardiovascular damage in the long-term (more than 30 days), and might be related to the increased mortality rate found in diabetic rats after a long-term myocardial infarction (90 days) [43]. Therefore, short-term hyperglycemia may protect the heart against an ischemic insult, reducing cardiovascular sympathetic modulation and preserving ventricular function.

### Cardiac oxidative stress profile

To investigate the intracellular mechanisms contributing to the attenuation of sympathetic modulation, we evaluated the oxidative stress profile in the LV tissue. Myocardial infarction in normoglicemic rats promoted a reduction of GPx activity, an increase in superoxide anion, lipoperoxidation and protein carbonilation, as well as a decrease in GSH/GSSG (p < 0.05 vs. C group), thus showing a worsened redox balance after ischemia. Therefore, these results combined may contribute to the imbalance of survival factors after myocardial infarction, through the increase in proapoptotic protein expression [44].

Hyperglycemia or myocardial infarction leads to oxidative stress and several other another abnormalities, causing changes in cellular signaling. These diabetes-mediated biochemical anomalies cross-interact in a complex interplay. These changes also lead to an alteration of transcriptional and post-transcriptional machinery, resulting in altered production of vasoactive and cardioactive factors by augmented production of reactive oxygen species such as superoxide (O_2_^−^), hydroxyl (OH^−^) and non-radicals, consequently reducing antioxidant defenses [14,45]. Therefore, these findings seem to depend on the period of hyperglycemia exposition. In this sense, STZ-induced diabetes in aged spontaneously hypertensive rats showed that the cardiac remodeling is associated with increased oxidative stress and activated fetal gene program [46].

In fact, short-term hyperglycemia initiates compensatory mechanisms. In this sense we demonstrated for the first time a statistically significant increase in antioxidant enzyme activity (CAT and GPx) and decrease in superoxide anion levels, lipoperoxidation and protein carbonilation, along with an increase in GSH/GSSG in diabetic animals after myocardial infarction (DMI group) when compared to normoglycemic animals (MI group) (p < 0.05). This indicates a reduced LV oxidative stress in infarcted animals previously exposed to hyperglycemia. These changes may be related to our previous findings, which demonstrated improvements in the ventricular response and infarcted area, and increased CAT activity and mortality rate in diabetic rats evaluated for short- term hyperglycemia (14 days after diabetes induction and exposed to 7 days of ischemic injury) [13].

Antioxidant converts reactive oxygen and nitrogen species to the role of second messenger involved in cell signalling. The adaptative capacity of the cells is altered by the balance between death or survival signals, converging at the level of the mitochondria, with distinct pathophysiologic consequences that extends the period of injury. Altered glucose flux, mitochondrial derangements and nitric oxide synthase uncoupling in the presence of decreased antioxidant defense and impaired pro survival cell signaling may render the diabetic myocardium more vulnerable to injury, remodelling and heart failure [47]. Additionally, ROS-stimulated activation of PERK (protein kinase RNA- like ER kinase) signaling pathway plays a key role in ROS-medicated endoplasmic reticulum stress-induced myocyte apoptosis in diabetic cardiomyopathy [48].

Finally, decreased cardiovascular sympathetic modulation may contribute to increase the expression of cell survival factors and to reduce oxidative stress, thus decreasing akinetic area and improving systolic function in diabetic infarcted animals. In this sense, the Person correlations between cardiac and vascular sympathetic modulation and LV oxidative stress evaluations (CAT, superoxide anion and GSH/GSSG) found in the present study reinforce the role of the autonomic nervous system in the management of cellular responses to ischemia.

In conclusion, our data suggest that hyperglycemia initiated compensatory mechanisms that may provide protection against ischemia, as demonstrated by increased antioxidants, decreased pro-oxidants and tissue damage associated with improvements in redox balance and cardiovascular sympathetic modulation. Taken together, these positive changes promoted a significant reduction in infarcted area, thus enabling cellular survival with improvement of systolic function.

## Abbreviations

AP: Arterial pressure
BP: Blood pressure
CAT: Catalase
C: Control
D: Diabetic
DMI: Diabetic myocardial infarcted
DAP: Diastolic arterial pressure
DNPH: Dinitrofenylhydrazyne
GPx: Glutathione peroxidase
HR: Heart rate
H_2_O_2_: Hydrogen peroxide
HIF-1: Hypoxia-inducible factor
HF: High-frequency band
LV: Left ventricle
LF: Low-frequency band
MDA: Malondialdehyde
MAP: Mean arterial pressure
MI: Myocardium infarcted
GSSG: Oxidized glutathione
PMSF: Phenylmethylsulfonyl fluoride
HRV: Power of heart rate
BPV: Power of systolic blood pressure
PI: Pulse interval
ROS: Reactive oxygen species
GSH: Reduced glutathione
PBS: Saline phosphate buffer
STZ: Streptozotocin
SOD: Superoxide dismutase
LF/HF: Sympathovagal balance
SBP: Systolic blood pressure
TBARS: Thiobarbituric acid reactive substances
RMSSD: The square root of the mean of the sum of the squares of differences between adjacent intervals
VAR-PI: Variance of pulse interval
